# P-1918. Incidence of viral rebound in hospitalized COVID-19 patients

**DOI:** 10.1093/ofid/ofae631.2078

**Published:** 2025-01-29

**Authors:** Tzu-Ping Weng, Lingshan Syue, Mingchi Li, Nan-Yao Lee

**Affiliations:** National Cheng Kung University Hospital, Tainan, Tainan, Taiwan; National Cheng Kung University Hospital, Tainan, Tainan, Taiwan; National Cheng Kung University Hospital, Tainan, Tainan, Taiwan; National Cheng Kung University Hospital, Tainan, Tainan, Taiwan

## Abstract

**Background:**

Viral rebound, characterized by a resurgence of viral activity after initial suppression, presents a potential threat to infection control within healthcare settings. We aimed to investigate the incidence of viral rebound in patients hospitalized for COVID-19.

**Methods:**

The retrospective study included symptomatic adults admitted for COVID-19 to a tertiary hospital in Taiwan during 1 January 2022 to 31 December 2022. Viral rebound was defined as an increase of △Ct ≥ 5 units or a new positive test after having tested negative during 10-30 days after COVID-19 diagnosis. The indication of antiviral agent was evaluated by physician according to initial severity and risks to develop severe COVID-19. The patients with prior COVID-19 infections, receipt of ≥ 2 antiviral agents, incomplete treatment course of antiviral agent, or active problem for hospitalization other than COVID-19 were excluded.

**Results:**

Of the included 354 participants, 26 received Paxlovid, 98 received molnupiravir, 54 received 3-day remdesivir, 101 received 5-day remdesivir, and 84 didn’t receive any antiviral agent. The incidence of viral rebound was higher in patients not receiving antiviral agent (n=75) compared to those who received either antiviral therapy (n=279) (17.3% vs. 11.5%, p=0.18). Additionally, the duration from COVID-19 diagnosis to onset of viral rebound was similar between the two groups (16.2 days vs. 14.3 days, p=0.20). Among the 148 with severe COVID-19, viral rebound was higher in patients without antiviral agent exposure (n=47) compared to those who received 5-day remdesivir (n=101) (21.3% vs. 15.8%, p=0.18). In 206 with non-severe disease, no significant differences were found in the incidence of viral rebound between patients who received Paxlovid (n=26) and molnupiravir (n=98) (11.5% vs. 7.1%, p=0.44).

**Conclusion:**

Our study highlights the risk of viral rebound in hospitalized COVID-19 patients. Patients without antiviral treatment had a higher rebound incidence. Among severe cases, antiviral therapy reduced rebound rates, underscoring its importance. Interestingly, antiviral choice did not significantly affect rebound incidences in non-severe cases. These findings stress the crucial role of antiviral therapy in infection control strategies.

**Disclosures:**

All Authors: No reported disclosures

